# Regional Homogeneity Abnormalities Affected by Depressive Symptoms in Migraine Patients without Aura: A Resting State Study

**DOI:** 10.1371/journal.pone.0077933

**Published:** 2013-10-16

**Authors:** Dahua Yu, Kai Yuan, Ling Zhao, Fanrong Liang, Wei Qin

**Affiliations:** 1 Information Processing Laboratory, School of Information Engineering, Inner Mongolia University of Science and Technology, Baotou, Inner Mongolia, China; 2 Life Sciences Research Center, School of Life Science and Technology, Xidian University, Xi’an, Shaanxi, China; 3 The 3rd Teaching Hospital, Chengdu University of Traditional Chinese Medicine, Chengdu, Sichuan, China; Laureate Institute for Brain Research and The University of Oklahoma, United States of America

## Abstract

**Background:**

Bidirectional relationship between migraine and depression suggests that there might be some etiological risk factors shared. However, few studies investigated resting state abnormalities affected by depressive symptoms in migraine patients without aura (MWoA).

**Materials and Methods:**

According to their self-rating depression scale (SDS) score, MWoA were divided into twenty in the SDS (+) (SDS > 49) group and 20 in the SDS (−) (SDS ≤ 49) group. Regional homogeneity (ReHo) method were employed to assess local features of spontaneous brain activity between 1) all MWoA and healthy controls, 2) each subgroup and healthy controls, and 3) SDS (−) group and SDS (+) group.

**Results:**

Compared with healthy controls, decreased ReHo in similar regions were shown in the MWoA group and subgroups. It is noteworthy that the caudate showed increased ReHo in the SDS (−) group compared with healthy controls and the SDS (+) group. Moreover, the average ReHo values of the caudate in SDS (−) group were significantly positively correlated with duration of migraine.

**Conclusions:**

Our results suggested that ReHo patterns in migraine patients may be affected by depressive symptoms and serve as a biomarker to reflect depression severity in MWoA.

## Introduction

As a primary headache disorder, migraine may be associated with a number of physiological and emotional stressors and even increase the risk of psychiatric disorders with a variety of psychological characteristics such as depressive symptoms [1]. A bidirectional relationship between migraine and depression suggests that there might be some etiological risk factors shared between migraine and depression [1,2]. However, the spontaneous fluctuations of neuronal activity at the baseline state affected by depressive symptoms in migraine patients remains unclear. 

As a noninvasive access, the spontaneous fluctuations of neuronal activity during the resting state may influence task performance in real life and revealed a reduction in integrity and efficiency of disease-relevant networks, which may be beneficial for the understanding of disease states [3]. Recently, Regional homogeneity (ReHo) analysis was developed as a data-driven method to analyze the synchronization of the blood oxygen level-dependent (BOLD) signal among neighboring voxels of the brain during the resting state [4]. It had been shown to advance the understanding of the complexity of brain function and to complement the functional connectivity analysis during the resting state and task-related fMRI studies [4]. 

Previous resting state studies revealed abnormal ReHo in migraine and depressive patients respectively [5–14]. On one hand, migraine patients without aura (MWoA) showed a significant decrease in ReHo values in the right rostral anterior cingulate cortex (rACC), the prefrontal cortex (PFC), the orbitofrontal cortex (OFC) and the supplementary motor area (SMA) compared with healthy controls [5]. Additionally, ReHo values were negatively correlated with the duration of disease in the right rACC and PFC [5]. On the other hand, ReHo abnormalities in patients with depression were also investigated. Compared with healthy controls, decreased ReHo values were found mainly in the OFC, fusiform gyrus, ACC, posterior cingulate cortex, lentiform nucleus, insula, caudate, posterior fusiform gyrus (PFG) the inferior frontal area, thalamus, inferior parietal lobule (IPL) and precuneus, cerebellum, superior frontal cortex (SFC), and increased ReHo values were also reported mainly in the right parahippocampal gyrus and temporal gyrus in patients with depression [6–14]. These findings supported the assertion that repeated migraine attacks or depression symptoms over time would result in selective damage to ReHo in several brain regions during the resting state. However, to the best of our knowledge, few studies focused on the specific feature of ReHo during the resting state affected by depressive symptoms in MWoA. 

To account for this aim, MWoA were divided into two groups according to their self-rating depression scale (SDS) score [15] as in our previous study [2], including the SDS (+) (SDS > 49) group and SDS (−) (SDS ≤ 49) group [15]. The ReHo method was employed to investigated the BOLD signal of the brain during the resting state among the whole MWoA, SDS (−) group, SDS (+) group and healthy controls in the present study [4].

## Methods

### Subjects

This study was approved by the Medical Ethics Committee of the West China Hospital at Sichuan University. MWoA were screened following the diagnostic criteria of the International Headache Society [16]. Age- and gender-matched healthy controls that had no family members who either suffered from any type of headache or depression disorder were enrolled. The exclusion criteria for both groups were: 1) any physical illness such as a brain tumor, hepatitis, diabetes or epilepsy as assessed according to clinical evaluations and medical records; 2) any psychiatric disease; 3) alcohol, nicotine or drug abuse; 4) pregnancy or menstrual period in women; 5) not being right-handed as measured by the Edinburgh Handedness Inventory ; 6) organic brain defects on T1 or T2 images (examined by two experienced radiologist); 7) claustrophobia and 8) any anti-depressant treatment.

At last, forty MWoA (11 males and 29 females, aged 22–57 years, mean age, 35.9±10.3 years) and 40 age- and gender-matched healthy controls (10 males and 30 females, aged 21–54 years, mean age, 33.2±9.5 years) were recruited in our study. All participants signed the informed consent after the experimental procedure was fully explained. Prior to scanning, urine drug screening was performed on all subjects to exclude the possibility of substance abuse. Attack frequency in the past 4 weeks and duration of migraine attacks was also rated. All of patients were not either having a migraine precipitated during or on the day following the scan or a migraine attack at least 72 hours prior to scanning [17]. In addition, depression symptoms of all participants was evaluated by the SDS test [15]. The SDS consists of 20 items presented in a 4-point multiple-choice format with a scale of 1 to 4. The scores ranged from 20 to 80 with higher scores indicating increased level of depressive symptoms. The four possible outcomes were as follows: 20–49 normal range, 50–59 mildly depressed, 60–69 moderately depressed, 70 and above severely depressed [18]. According to the SDS scores, the MWoA were divided into two groups including the SDS (−) group (7 males and 13 females, aged 22–57 years, with a mean age: 33.6±11.7 years, SDS score under 49 with a mean score: 39.1±5.0) and the rest were assigned to the SDS (+) group (4 males and 16 females, aged 22–55 years, with a mean age: 38.2±8.4 years, SDS score 60.3±6.6). The mean SDS score in healthy controls was 37.6±4.0. Average pain intensity was rated by the visual analogue scale (VAS) on a 0-10 scale from attacks in the past 4 weeks, which was 4.8±1.4 in the SDS (−) group and 5.5±1.9 in the SDS (+) group. The clinical and demographic characteristics of participants are shown in Table 1. 

**Table 1:** The clinical and demographic characteristics of participants (means ± standard deviations).

Clinical details	Controls (n=40)	MWoA (n=40)	SDS (−) (n=20)	SDS (+) (n=20)
Age (years)	33.2±9.5	35.9±10.3	33.6±11.7	38.2±8.4
*	vs MWoA: p = .229	vs controls: p = .229	vs controls: p = .884	vs controls: p = .070
*	vs SDS (−): p = .884	vs SDS (−): p = .402	vs MWoA: p = .402	vs MWoA: p = .402
*	vs SDS (+): p = .070	vs SDS (+): p = .402	vs SDS (+): p = .148	vs SDS (−): p = .148
Gender	16F, 4M	29 F, 11 M	13F, 7M	16F, 4M
*	vs MWoA: p = .444	vs controls: p = .444	vs controls: p = .212	vs controls: p = 1.00
*	vs SDS (−): p = .212	vs SDS (−): p = .532	vs MWoA: p = .532	vs MWoA: p = .532
*	vs SDS (+): p = 1.00	vs SDS (+): p = .532	vs SDS (+): p = .280	vs SDS (−): p = .280
Duration of migraine (years)	—	10.8±7.1	12.4±8.0	9.2±5.8
*	—	vs SDS (−): p = .402	vs MWoA: p = .402	vs MWoA: p = .402
*	—	vs SDS (+): p = .402	vs SDS (+): p = .149	vs SDS (−): p = .149
Attack frequency (times in past four weeks)	—	5.2±3.6	4.2±2.7	6.3±4.1
*	—	vs SDS (−): p = .293	vs MWoA: p = .293	vs MWoA: p = .293
*	—	vs SDS (+): p = .293	vs SDS (+): p = .071	vs SDS (−): p = .071
VAS (0-10)	—	5.1±1.7	4.8±1.4	5.5±1.9
*	—	vs SDS (−): p = .463	vs MWoA: p = .463	vs MWoA: p = .463
*	—	vs SDS (+): p = .463	vs SDS (+): p = .205	vs SDS (−): p = .205
SDS scores	37.6±4.0	49.7±12.2	39.1±5.0	60.3±6.6
*	vs MWoA: p = .000	vs controls: p = .000	vs controls: p = .496	vs controls: p = .000
*	vs SDS (−): p = .496	vs SDS (−): p = .000	vs MWoA: p = .000	vs MWoA: p = .000
*	vs SDS (+): p = .000	vs SDS (+): p = .000	vs SDS (+): p = .000	vs SDS (−): p = .000

* Pairwise t-test for each group combination with multiple comparison correction (LSD, Least Significant Difference).

Abbreviation: MwoA, migraine patients without aura; F, female; M, male; VAS, visual analogue scale; SDS, self-rating depression scale; SDS (+): migraine patients without aura with high depressive symptoms, self-rating depression scale (SDS) scores > 49; SDS (−): migraine patients without aura with low depressive symptoms, SDS scores ≤ 49.

### Data acquisition

All data were acquired on a 3T Siemens magnetic resonance (MR) scanner (Allegra; Siemens Medical System) at the Huaxi MR Research Center, West China Hospital at Sichuan University, Chengdu, China. The heads of the subjects were positioned carefully with comfortable support to reduce head motion. Prior to the functional run, T1 and T2 weighted images for each subject were acquired to exclude the possibility of clinically silent lesions and were examined by two expert radiologists. The resting state functional images were obtained with an echo-planar imaging (EPI) sequence (30 contiguous slices with slice thickness = 5 mm, TR = 2,000 ms, TE = 30 ms, flip angle = 90°, FOV = 240×240 mm^2^, data matrix = 64 ×64, and total volumes = 180). Subjects were instructed to keep their eyes closed, not to think about anything, and to stay awake during the entire 6 minute functional scan. After the scan, each subject was asked whether s/he remained awake during the whole procedure. 

### Data analysis

After an inspection of the quality of raw functional images, all of the data preprocessing procedures were performed in SPM5 (http://www.fil.ion.ucl.ac.uk/spm), including slice timing, realignment, and normalization. The first five volumes were discarded to ensure stable magnetization and allow participants to adapt to the scanning environment. The images were corrected for the acquisition delay between slices, aligned to the first image of each session for motion correction, and spatially normalized to the standard MNI template in SPM5. No subjects had head motion exceeding 1 mm of movement or 1° of rotation in any direction. A band-pass filter (0.01 Hz < f < 0.08 Hz) was applied to remove physiological and high frequency noise. To reduce the effects of head motion and non-neuronal BOLD (heart rate and respiration changes), nine nuisance signals (the global mean, white matter, and cerebrospinal fluid signals and 6 motion parameters) were regressed out as nuisance covariates.

By using the Resting-State fMRI Data Analysis Toolkit (REST, by SONG Xiao-Wei et al., http://www.restfmri.net), Kendall’s coefficient concordance (KCC) was used to evaluate regional homogeneity [4]. A whole brain mask was used to remove non-brain tissues. Individual ReHo maps were generated by assigning each voxel a value corresponding to the KCC of its time series with its nearest 26 neighboring voxels [4].The individual ReHo maps were standardized by their own mean KCC within the mask . Then, a Gaussian kernel of 4 mm full width at half-maximum (FWHM) was used to smooth the images for the aim to reduce noise and residual differences [19]. 

Statistical analysis of functional images was performed in SPM5 (http://www.fil.ion.ucl.ac.uk/spm). For the whole MWoA group and healthy controls, a one-sample *t*-test (*p* < 0.05, family-wise error (FWE) correction) was performed to extract the ReHo results across the subjects within each group. Then, a two-sample *t*-test was applied to compare the ReHo results 1) between all healthy controls and all MWoA, 2) between each subgroup (the SDS (−) group and SDS (+) group) and controls, and 3) between the SDS (−) group and SDS (+) group to examine differences in ReHo (*p* < 0.05, FWE corrected). Finally, to further investigate the association between the ReHo abnormality and the duration in MWoA, Pearson’s correlation analyses were carried out. The average ReHo values in the regions of interest (ROIs) were extracted, averaged and correlated with the duration of MWoA. Distributions of age, gender, duration, attack frequency, VAS and SDS scores among groups and correlation analysis were analyzed using SPSS statistical analysis software (version 17.0, SPSS Inc., Chicago, Illinois) and MATLAB (2010b, Natick, Massachusetts; The Mathworks Inc.).

## Results

**Figure 1:**
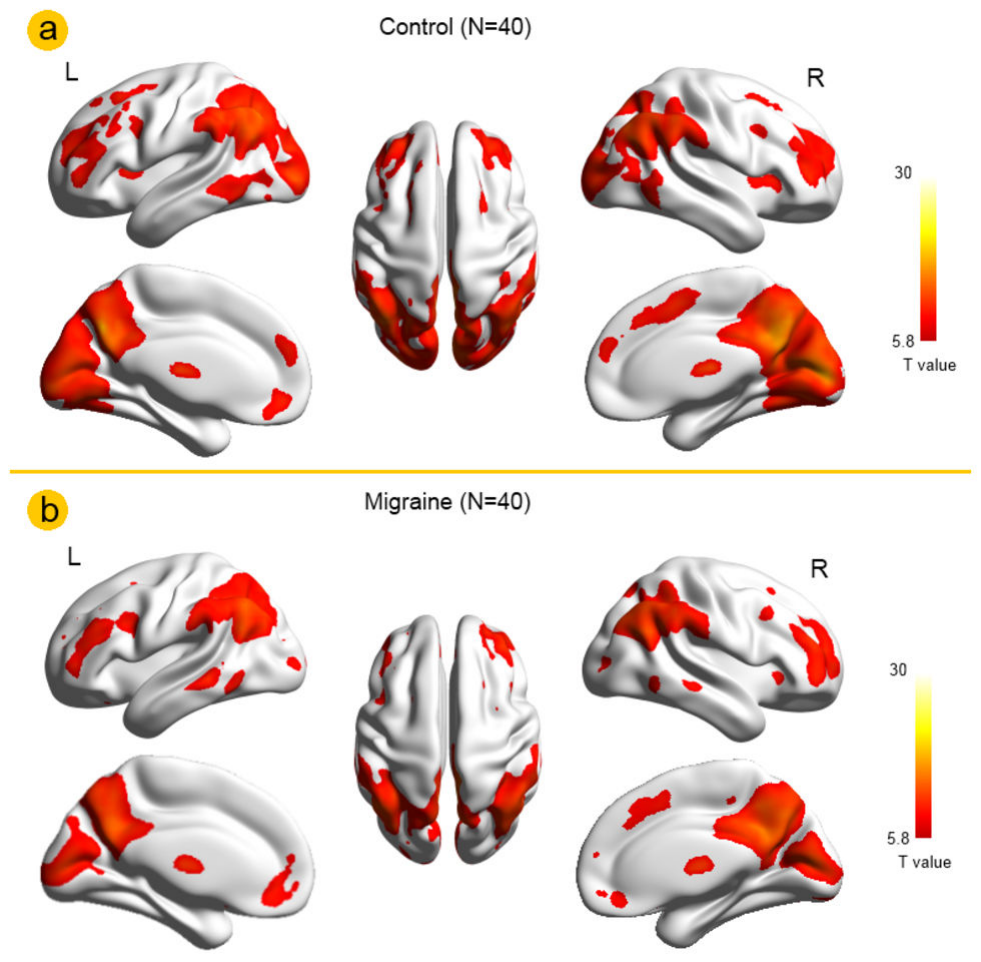
Regional homogeneity (ReHo) results within the migraine patients without aura (MWoA) and healthy controls. (a) MwoA ReHo map, (b) healthy controls ReHo map (*p* <0.05, FWE corrected).

**Figure 2:**
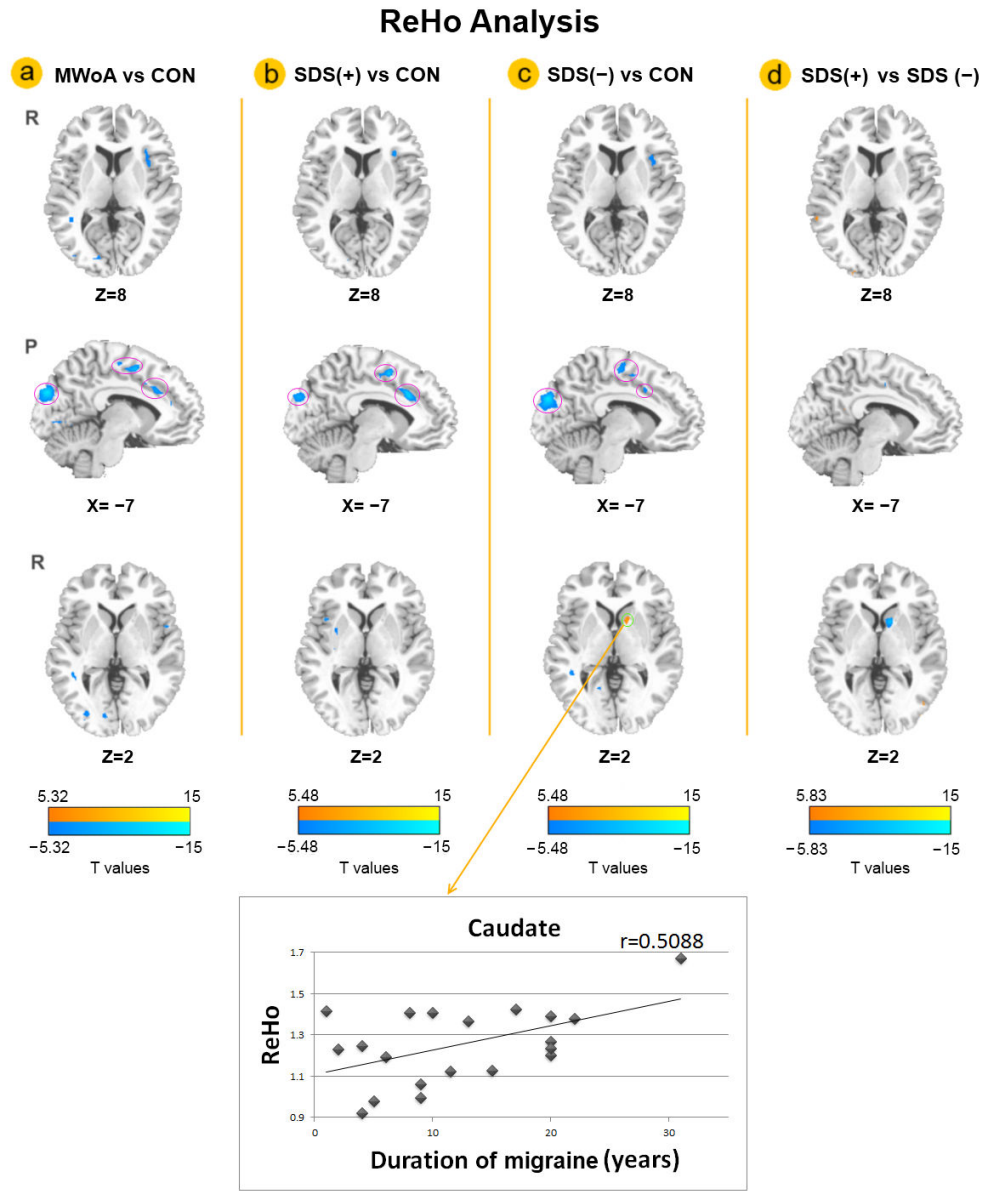
Abnormal regional homogeneity (ReHo) changes between groups. Compared with healthy controls, decreased ReHo in the insula, the rostral anterior cingulate cortex (rACC), the supplementary motor area (SMA) and the cuneus were shown similarly in the whole migraine patients without aura (MWoA) group (Figure 2.a), SDS (+) group (Figure 2.b) and SDS (−) group (Figure 2.c) (p < 0.05, FWE corrected), which was indicate by a cool color. It is noteworthy that the caudate showed increased ReHo in the SDS (−) group compared with healthy controls (at the bottom of Figure 2.c shown in a warm color), and compared with the SDS (+) group (at the bottom of Figure 2.d in a cool color) (p < 0.05, FWE corrected). As indicated by the straight lines with the arrow, the average ReHo values of the caudate in Figure 2.c was significantly positively correlated with duration of migraine in the SDS (−) group separately. (SDS (+) group: migraine patients without aura with high depressive symptoms, self-rating depression scale (SDS) scores > 49; SDS (−) group: migraine patients without aura with low depressive symptoms, SDS scores ≤ 49.)

As shown in Table 1, except for the SDS scores, the clinical and demographic characteristics of each groups revealed no significant difference. ReHo results within the MWoA and healthy controls are shown in Figure 1 (*p* < 0.05, FWE corrected). The major regions of the default mode network (DMN) exhibited significantly higher ReHo values than other brain regions during the resting state, including the medial prefrontal cortex (MPFC), the posterior cingulate cortex (PCC), the medial temporal lobe, the precuneus and IPL (Figure 1 a, b). The results of the two-sample *t*-test are shown in Figure 2. Compared with healthy controls, decreased ReHo values located in the insula, the rACC, the SMA and the cuneus were similarly shown in the whole MWoA group, SDS (−) group and SDS (+) group (*p* < 0.05, FWE corrected) (Figure 2 a, b, c), while no brain region with increased ReHo values was observed in the whole MWoA group and SDS (+) group (Figure 2 a, b). Furthermore, compared with healthy controls, increased ReHo values in the caudate were shown in the SDS (−) group (*p* < 0.05, FWE corrected) (Figure 2 c). As shown in Figure 2 d, compared with the SDS (+) group, the SDS (−) group also showed increased ReHo values in the caudate (*p* < 0.05, FWE corrected). In addition, the correlation results demonstrating the average ReHo value of the caudate in Figure 2.c was positively correlated with duration of migraine in the SDS (−) group (r = 0.5088, *p* = 0.022) (the bottom of Figure 2 as indicated by the straight lines with the arrow). 

## Discussion

During the resting state, correlated spontaneous fluctuations occur within spatially distinct areas, some of them are cortical and subcortical regions which are functionally related and consist of the human brain’s intrinsic functional networks, whose variations may influence task performance in real life [3]. ReHo has been proven to be sufficient in detecting local features of spontaneous brain activity observed in specific regions [4]. Abnormal ReHo values in related brain regions were reported by previous migraine or depression studies during the resting-state [5–13]. However, few studies investigated ReHo abnormalities affected by depressive symptoms in MWoA. 

In the present study, ReHo analysis was used to specify the resting state properties affected by depressive symptoms in MWoA. First of all, a one sample t-test revealed several specific brain regions with higher regional homogeneity during the resting state than the others, which constitute a network supporting a default mode of brain function. The DMN consisted of a series of brain regions involving retrieval and manipulation of past events or problem solving and future plans, which exhibit synchronized low frequency oscillations at resting-state [20]. We identified the DMN exhibiting significantly higher ReHo values than other brain regions in each group during the resting state (Figure 1 a, b), i.e. the medial temporal lobe, PCC, precuneus, MPFC, and IPL . In our current study, this pattern is similar in the whole migraine patients group and healthy controls and is in line with previous studies [4,5].

### ReHo abnormalities related with migraine

Similar as our previous findings [5], the findings in the present study validated decreased ReHo abnormalities of the rACC, the SMA, the insula and the cuneus consistently in the whole migraine patient group, SDS (−) group and SDS (+) group compared with the healthy control group (Figure 2 a, b, c). The consistent results may suggest that the decreased ReHo in these regions is related with migraine. These brain regions with decreased ReHo in MWoA were similar to the brain regions reported in structural [21,22] and functional studies [5,17,22–24] in migraine, which were mainly involved in pain-related processing [22]. In particular, decreased ReHo values in the rACC and the SMA were reported in a previous study [5]. The rACC is principally associated with pain responses and endogenous pain control which is mediated by endogenous opioid systems [25]. Moreover, the SMA is involved in executive-control , pain anticipation and affective component of pain [26]. Another region which is well known to be involved in pain processing is the insula [27]. The insula was consistently activated in PET studies [28] and task-related fMRI studies of migraine [17,23,24]. We speculated that decreased ReHo values of these regions may be related with structural and functional impairments in MwoA and possibly suggest efficiency reduction in pain processing in MWoA. 

### Abnormal ReHo changes in the caudate in MWoA

It is interesting that increased ReHo in the caudate showed only in the SDS (−) group in our study. In more detail, the caudate showed increased ReHo in the SDS (−) group compared with healthy controls (Figure 2 c), and the SDS (+) group (Figure 2 d), while no abnormal ReHo in the caudate was shown in the SDS (+) group or whole MWoA group compared with healthy controls. As an important part of the basal ganglia, the caudate is involved in multisensory integration, pain processing and depression [7,29]. The increased ReHo of the caudate was observed in the SDS (-) group compared with healthy controls, which might be related to migraine. The important role of the caudate in migraine neuropathology has been investigated [30]. In addition, abnormal activation in the caudate was also reported in previous pain-related studies, which may suggest that the caudate is a part of the pain modulatory system [31]. Furthermore, the results of the correlation analysis demonstrated that the average ReHo values of the caudate in the SDS (−) group compared with healthy controls were positively correlated with duration in the SDS (−) group (Figure 2.c). It may validate the association between the caudate and migraine pathology.

Meanwhile, our results validated the abnormal ReHo values in the caudate in the comparison between the SDS (−) group and the SDS (+) group. Considering the result of the SDS (+) group and SDS (−) group compared with health controls, it may be explained by the increase of ReHo in the SDS (−) group compared with the SDS (+) group. The comparison between the SDS (+) group and SDS (−) group might be related with the depression symptoms in MWoA. One of the possible explanations is that the effects from migraine and depression symptoms might be counteracted in the caudate, which may be related with seemingly normal ReHo in the caudate of the SDS (+) group. As a key brain structure involved in the regulation of cognition and mood, the decreased Reho values in the caudate were found in depression [14] and treatment-refractory depression [7]. In previous studies of depression, the caudate showed reduced gray matter volume [32] and impaired metabolism [33]. Antidepressant treatment also showed a reduction in task-related activity in the caudate [34]. Our results might help us to understand how antidepressants help to reduce the severity of migraine or prevent migraine. Abnormalities in the caudate were also associated with a higher prevalence of depression or depressive symptoms in Huntington disease [35] and Parkinson disease [36]. All of these findings may suggest that the caudate may be involved in the etiology of depressive symptoms, not only in depressive disorder itself but also in other disorders with a depressive component in their symptomatology. Compatible with these previous studies in migraine and depression, the effects from migraine and depression symptoms might be counteracted in the caudate, which showed no abnormal ReHo in the SDS (+) group or whole MWoA group compared with healthy controls.

### Limitation

The relationship between migraine and depressive symptoms may be bi-directional, or may result from changes in correlated latent variables. The present study is limited in the cross-sectional design. The inference of causality is not possible to postulate between current illness severity and abnormal ReHo or between migraine and depression symptoms. Further longitudinal studies or analysis of variance studies would improve the understanding of it. Furthermore, it is always a great challenge as we attempt to explain underlying neural mechanism of ReHo. Our findings might provide another fragment of migraine pathophysiology, which is needed more work to improve it.

## Conclusions

In the current study, we employed a ReHo method to assess local features of spontaneous brain activity affected by migraine and depressive symptoms in MWoA during the resting state. We suggested that the ReHo changes in the caudate may provide more scientific evidence for the role in the pathology of migraine, which may serve as a sensitive biomarker to reflect depression severity and disease progression of migraine without aura. However, a more comprehensive experimental design is needed to reveal the accurate roles of these ReHo abnormalities in the pathology of migraine with depression symptoms. We hope that our results could provide more evidence during the resting state in migraine patients and improve the understanding of migraine pathophysiology.
